# Harnessing macrogenomics to study changing marine ecosystems under global climate change

**DOI:** 10.1098/rspb.2026.0357

**Published:** 2026-10-07

**Authors:** Nicolas Dussex, Leif Andersson, Ulf Bergström, Kerstin Johannesson, Andreas Wallberg

**Affiliations:** ^1^ Department of Population Analysis and Monitoring, Swedish Museum of Natural History, Stockholm, Sweden; ^2^ Department of Medical Biochemistry and Microbiology, Uppsala University, Uppsala, Sweden; ^3^ Department of Veterinary Integrative Biosciences, Texas A&M University, College Station, TX, USA; ^4^ Department of Aquatic Resources, Swedish University of Agricultural Sciences, Uppsala, Sweden; ^5^ Department of Marine Sciences, University of Gothenburg, Tjärnö Marine Laboratory, Strömstad, Sweden

**Keywords:** genomics, evolution, conservation, global warming, macrogenomics

## Abstract

Climate change is reshaping marine ecosystems at an accelerating pace. Predicting its impacts on biodiversity and ecosystem functions is therefore crucial to developing effective conservation strategies. Integration of genomic tools, habitat modelling and simulations offers a transformative approach to forecast these impacts. Yet, current frameworks remain limited to single-species analyses. In this review, we discuss future directions in the emerging field of macrogenomics, the analysis of large-scale genomic data across species and time, and show how its integration with environmental data can be applied to understand past and predict future climate-driven ecological shifts across entire marine communities. We demonstrate how applying a macrogenomic framework can provide insights into future responses of marine ecosystems under global climate change by integrating multi-species demographic analyses, metagenomics and signatures of adaptation associated with past climate fluctuations. We also show how macrogenomics combined with predictive approaches can be used to forecast cascading effects of species fluctuations on ecosystem structure and potentially identify ecosystem tipping points. Ultimately, coupling empirical and prediction data offers a powerful approach for ecosystem-based management, improving our ability to predict adaptive responses and extinction risks at relevant scales, and equipping managers and policymakers with actionable insights to prioritize biodiversity conservation strategies.

## 1. Introduction

Global warming is severely affecting marine ecosystems, inducing shifts in species and interactions, changes in reproductive cycles, increased selective pressures on physiological functions and local or global species loss [1]. While some species may adapt, the impacts on marine ecosystems are likely to be severe and potentially irreversible. Species distributions will shift as species track ideal environmental conditions [2]. Cold-adapted species may be replaced by warm-adapted ones [3], potentially triggering cascading effects on ecosystem structure and functioning [4], such as mesopredator release (e.g. [5]). Similarly, the disappearance of piscivorous fish can lead to coastal ecosystems being dominated by filamentous algae, reduced grazer populations, loss of seagrass and increased anoxic sediments [6].

Yet, the effects of global warming on marine ecosystems have mostly been explored experimentally or in the wild over a few generations. We thus lack data on these effects over longer timescales (millennia), which could be invaluable for predicting future demographic and adaptive responses. Moreover, it remains unclear how climate and environmental changes will affect species dynamics and reshape whole ecosystems.

Examining species' responses to contemporary and past climatic fluctuations provides important insights into how future warming will impact ecosystems [6]. Next-generation sequencing (NGS) has transformed evolutionary biology, conservation and ecology, contributing significantly to studying species' dynamics and evolution through time and space. Demographic reconstructions [7] have proven essential for assessing the impacts of past climate fluctuations in terrestrial [8] and marine organisms [9] and for identifying the drivers of extinction. In addition, identifying genetically distinct populations and comparing their demographic trajectories can inform management decisions such as harvesting quotas [10]. Genomic analyses also reveal patterns and rates of adaptation to environmental variation, improving predictions of species' responses to climate change [11]. Genomics has also become central to conservation science, as genome-wide diversity is crucial for long-term viability and resilience, with intraspecific genetic variation (IGV) routinely estimated [12]. Finally, decreasing sequencing costs and expanding genomic consortia have broadened access to these approaches for rare and endangered species (e.g. [13]).

Several analytical frameworks assess species responses to environmental change, including space-for-time approaches [14], ancient and historical DNA (aDNA/histDNA) retrieved from degraded material such as subfossil bones or material from museum collections [15], environmental DNA (eDNA) and sedimentary ancient DNA (sedaDNA) obtained from water or soil samples [16], species distribution modelling (SDM) [17], seascape genomics [18–20] and genomic simulations [21]. Although many genetic or genomic studies have provided important insights into demographic and adaptive responses to climate change [11], most have focused on single species or taxonomic groups, overlooking species interactions, and thus limiting our understanding of ecosystem-level changes. Importantly, because species are embedded in trophic networks, genetic studies that ignore interspecific dependencies may underestimate ecosystem vulnerability to climate change and the risk of ecosystem collapse. By contrast, macrogenetics represents an upscaling of single-species studies, and instead leverages new or published genetic datasets of multiple species [22] to examine patterns of diversity across broad taxonomic, spatial and/or temporal scales, and to identify the predictors of intraspecific genetic variation. In the current biodiversity crisis, an ecosystem-based management approach is urgently needed. Macrogenetics supports this approach by moving beyond single-species analyses to provide insights into ecosystem response and vulnerability to environmental change.

In this review, we extend this approach to macrogenomics, which instead uses genomic data rather than data based on a few loci. Because Leigh *et al.* [22] already acknowledged the potential of metagenomics and metabarcoding, we propose a broader concept of macrogenomics that includes two conceptual frameworks: (i) genomic diversity across broad spatial, temporal, and taxonomic scales (e.g. population or community genomics at macroecological scales) and (ii) metagenomic variation from environmental samples. By demonstrating how combining genomic analyses with modelling can trace past ecological responses and forecast biodiversity shifts, we provide a forward-looking vision of how the emerging field of macrogenomics can transform marine conservation and ecosystem-based management under global climate change.

## 2. The potential of macrogenomics

A growing number of macrogenetic studies have sought to identify the predictors of genetic variation across diverse taxa and ecosystems (e.g. [23–26]). While transitioning from macrogenetics to macrogenomics introduces several challenges (e.g. computational power, data standardization requirements), the advantages clearly outweigh the drawbacks [22]. The growing affordability of shotgun-sequencing data enables more accurate characterization of genetic parameters, broader temporal and spatial resolution and community-level analyses combining whole genome and metagenomics data (i.e. genetic material recovered from environmental samples, such as soil, water or animal tissue) [27]. Yet, the number of macrogenomics studies is still relatively low, limiting our knowledge of the ecosystem-level effects of past climate change, such as shifts in trophic interactions and how these have reshaped communities. Furthermore, the mechanisms underlying adaptive responses to rapid global warming remain poorly understood, particularly regarding patterns of genome erosion and adaptations across species [25]. Studying demographic and genomic responses in a macrogenomics framework may thus help identify species most sensitive to shifts from their local environmental optima and thus may offer insights into ecosystem vulnerability and resilience to climate change [28].

In the era of big data genomics, leveraging published and newly sequenced data within a macrogenomic framework offers an unprecedented opportunity to better understand past and future climate impacts on biodiversity. Using multiple DNA sources (i.e. modern, historical, ancient, environmental and sedimentary) [27] provides an even broader view of temporal, ecosystem-wide changes than re-sequencing of modern data alone. Moreover, combining empirical data with simulations can improve our understanding of climate-driven ecosystem changes as well as help identify predictors of species responses and their capacity to retain genome-wide variation.

Out of the approximately 230 000 marine eukaryote species described (figure 1a), genomic datasets have been produced for more than 9 000 of them over the past 15 years (supplementary material, figure S1). However, even though large marine vertebrates are well represented in these databases, only 4% of all marine species have been sequenced overall, with algae and invertebrates being the most underrepresented given their taxonomic diversity (figure 1b). Genomic libraries include whole genome sequencing (WGS), reduced representation (e.g. amplicons, RNA-seq, RAD-seq) or metagenomics data (e.g. eDNA and sedaDNA) generated from modern, historical or ancient material (figure 1c and supplementary material), with modern WGS and RAD-seq libraries being predominant. The geographic origin of currently available genomic data is strongly biased towards the Northern Hemisphere, with the Arctic and Indian Oceans also notably undersampled (figure 1d). Moreover, fish and invertebrates are the only groups with over 100 species with at least 50 WGS libraries (figure 1e), indicating a need for more strategic sequencing to address gaps in geographic and taxonomic coverage.

**Figure 1.**
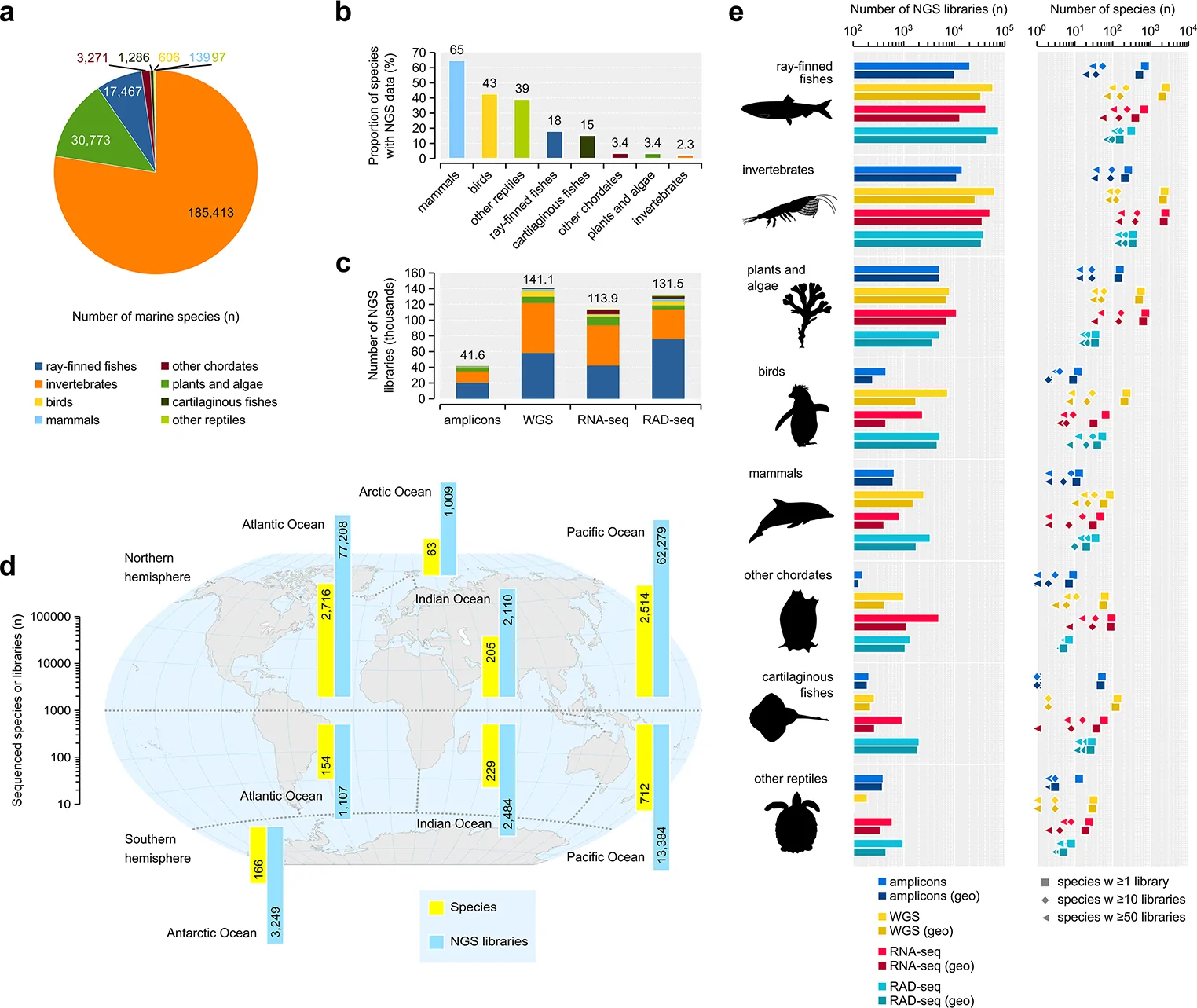
Published marine genomic NGS libraries (modern samples). NGS libraries in the NCBI Sequence Read Archive or the European Nucleotide Archive. (a) The combined numbers of marine species names in the WoRMS [29] and AlgaeBase [30] databases, divided by organism group (other reptiles = reptiles excluding birds; other chordates = chordates excluding the vertebrate groups in the figure). (b) Proportion of marine species with any NGS data (at least one NGS library). (c) Numbers of different types of NGS libraries obtained from modern material. WGS = whole genome sequencing, RNA-seq = RNA sequencing, RAD-seq = restriction-site associated DNA sequencing. The total number of libraries is given for each type. Colours as in (a). (d) The number of sequenced and geolocated species and libraries partitioned by hemispheres and oceans (log scale). Ocean map modified after [31]. (e) Left: number of modern NGS libraries per data type and group (geo = with geographic information). Right: number of species with *n* ≥ 1, *n* ≥ 10 or *n* ≥ 50 libraries per data type and group.

Although genomic datasets vary widely in sampling design, number of individuals and temporal or geographic resolution, different data types have successfully been combined and co-analysed in some single-species studies (e.g. [33]), and their rapidly increasing availability thus creates unprecedented opportunities for applications in multi-species and macrogenomic frameworks [22].

## 3. Applications of macrogenomics in marine ecosystems

The increasing availability of marine genomic resources, together with climate and species interaction datasets, provides an unprecedented opportunity to assess temporal marine ecosystem responses to global warming and environmental change.

We divide this review into four sections addressing key concerns and uncertainties outlined by the IPCC [34]: (i) species composition and ecosystem shifts, (ii) adaptive responses to environmental changes, (iii) ecosystem health based on genomic variation, and (iv) prediction of future ecosystem shifts.

### (a) Species composition and ecosystem shifts

Tracking past changes in species diversity and population size in relation to environmental variability is essential for predicting future responses of species and communities under climate change.

Subfossil data have been used to reconstruct past marine ecosystem dynamics driven by climate change, revealing fluctuations in species abundance [6]. For instance, Baltic Sea fossil assemblages have revealed diatom diversity shifts in response to salinity fluctuations and informed the timing of colonization by molluscs [35], whereas fossils have revealed the responses of the Great Barrier Reef to rapid sea level rise of the Holocene [36]. Historical and archaeological records [37] have also provided insights into fish stock fluctuations since the Stone Age (e.g. herring [38] and cod [39]). However, because of the spatio-temporally fragmented nature of fossil remains and risk of underestimation of taxonomic diversity [40], except for a few groups (e.g. planktonic foraminifera), sedaDNA has become an essential tool for reconstructing past marine ecosystems [41]. For instance, bulk-bone metabarcoding and sedaDNA have revealed major shifts in faunal, macroalgal, diatom and other microbial communities across both recent (20–75 000 years BP) and long (up to 2 million years BP) timescales, reflecting responses to warmer Pleistocene and Holocene conditions [42–45]. Additionally, the development of whole genome analyses for past demographic reconstructions (e.g. sequentially Markovian coalescent (SMC) methods [7] and linkage disequilibrium [46]) and detection of runs of homozygosity can reveal the timing and drivers of population size fluctuations over hundreds to hundreds of thousands of years from both modern DNA and aDNA [47,48].

A number of recent comparative demographic studies have studied species sensitivity to past climate shifts [49–52]. Yet, they have mostly focused on single taxonomic groups and have overlooked how climate change can indirectly alter species interactions, triggering trophic cascades and potential secondary extinctions. We argue that macrogenomic analyses, combining different data types (figure 2a), can examine both direct and indirect effects of climate change and provide insights into species-level fluctuations and ecosystem-level restructuring, potentially identifying key links within the ecosystem.

**Figure 2.**
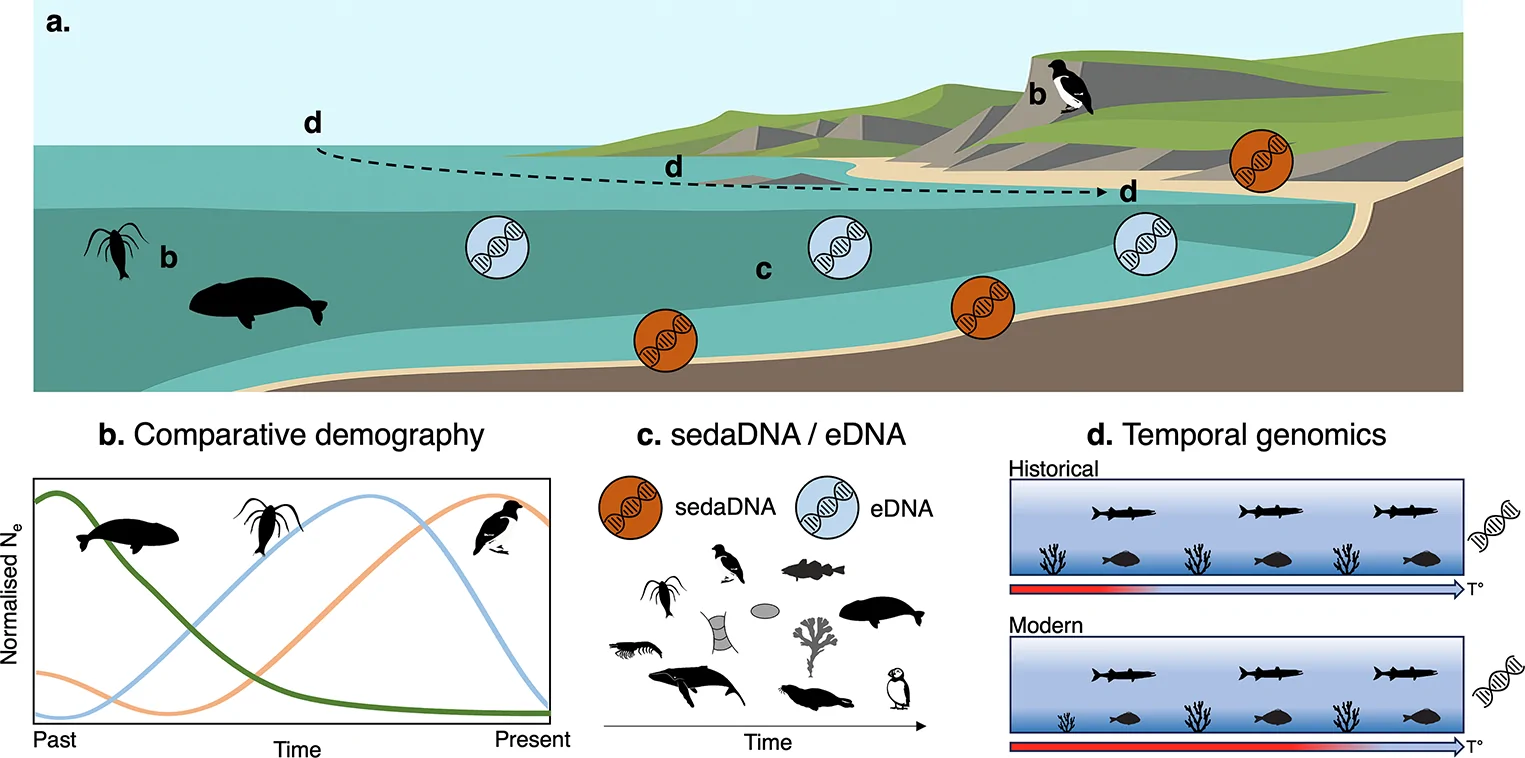
Usage of genomics to track temporal changes in marine ecosystems. (a) DNA sources in marine genomics. (b) Comparative demography can be used to test hypotheses on the sensitivity of species to climate change or on the cascading effects of species fluctuations (e.g. [32]). (c) Metagenomic data (i.e. sedaDNA and eDNA) can reveal temporal changes in relative species abundance of a broad range of organisms and can be coupled with demographic analyses (b) to track ecosystem shifts from deep (i.e. millions of years) to recent time (i.e. centuries). (d) Temporal genomics can detect changes in selective pressures in species distributed along an environmental gradient using temporally spaced genomes. Rising temperatures drive allele shifts across the gradient, affecting foundation species (e.g. coral), consumers (e.g. parrotfish) and predators (e.g. barracuda). Selection and population decline from physiological stress reduce diversity and fitness (e.g. size and reproductive period).

Since ecosystem shifts are logistically challenging to study experimentally (except for direct anthropogenic impacts such as overfishing or eutrophication, e.g. [53,54]) or owing to a fragmented record of past species abundance and population sizes, demographic reconstructions at the ecosystem level (e.g. small-scale: bay, tidal pool; broad-scale: Great Barrier Reef, Baltic Sea) and for multiple taxa, represent a valuable alternative, allowing the inference of ecosystem-level effects, cascading interactions and tipping points, linking vulnerable species to environmental changes (supplementary material, table S1). For instance, ecological modelling suggests that intensive hunting of North Atlantic bowhead whales reduced predation on zooplankton (i.e. copepods), favouring little auk population growth via competitive release [32]. Empirical genomic-based demographic reconstructions could thus be used to confirm a scenario of competitive release (figure 2b) or to test whether major demographic changes are instead mostly influenced by temperature fluctuations and glacial cycles (e.g. [55,56]). Considering trophic interactions alongside climatic variability is therefore crucial for reconstructing ecosystem shifts. The increasing availability of WGS data (figure 1; supplementary material, S1) makes such comparative demographic analyses timely.

Furthermore, combining species occurrence data inferred with metagenomics and species-specific demographic reconstructions could provide a more comprehensive view of ecosystem changes over time (figure 2c) and enable disentangling trophic interactions from climate effects [27]. SMC-based reconstructions combined with sedaDNA can inform on million-year to millennial-scale ecosystem shifts, whereas shorter timescale reconstructions (i.e. past 200 generations [46]) combined with historical time series of eDNA capture changes over the past centuries. For instance, eDNA from filter-feeding organisms preserved in museum collections has revealed biogeographic differences in marine biodiversity [57], temporal shifts in fish diversity [58] and invasion trajectories of non-native species over decades to centuries [59]. Combining sedaDNA and eDNA could reconstruct phytoplankton and algal communities spanning millennia to the present, shedding light on climate impacts on foundational marine food-web components.

Most eDNA and sedaDNA studies have focused on species detection or on shifts in species composition. However, recent work has demonstrated the potential of metagenomic data in population genomic analyses for a range of organisms (e.g. ctenophores and anchovies [60], penguins [61]), representing a promising innovation [62]. Consequently, these published datasets present an untapped resource for new analyses of genetic diversity in other organisms. Additionally, metagenomic studies of oral or gut microbiomes from temporally spaced samples could serve as proxies for dietary shifts, as demonstrated in humans [63], providing further insights into changes in marine ecosystems.

Ultimately, contextualizing these genomic signals (i.e. community shifts and past demography) within records of past climate shifts would help distinguish how much ecosystem change is driven by species-specific demographic fluctuations or trophic restructuring, yielding insights that could directly strengthen predictive frameworks for the future [16].

### (b) Adaptive responses to environmental change

Global climate change is putting increasing physiological pressure on marine organisms, disrupting cellular homeostasis, elevating metabolic demands and impairing growth and reproduction [64,65], making thermal tolerance, developmental rate and dispersal ability key targets of natural selection [1,66,67]. Analyses of population differentiation along environmental gradients (i.e. space-for-time approaches) have provided critical insights into adaptive responses to environmental change [14]. They have identified the genomic architecture underlying responses to salinity, temperature and habitat type across diverse taxa [68–72] and shown that few adaptive mutations or supergenes can reach near-fixation within decades to thousands of years [70,73,74], with life-history traits, dispersal ability and early life-stage sensitivity acting as key drivers of differentiation [68–70,74]. Such approaches have also revealed strong multi-species population structuring along environmental gradients (e.g. salinity in the Baltic Sea), indicating that adaptive chromosomal rearrangements can outweigh dispersal in shaping population differentiation [25], and showed widespread reductions in genetic diversity and loss of sexual reproduction that may constrain future adaptive capacity [75]. Additionally, natural or human-induced catastrophic events [76] have shown examples of rapid evolution within decades in threespine stickleback transitioning from marine to freshwater post-earthquake [77], and in rough periwinkle adapting to predator-free environments [74].

Comparative studies have also provided important insights into the functions under selection in changing environments, revealing both species-specific and convergent adaptive responses. For instance, multi-species seascape genomic studies have identified distinct adaptations to temperature, depth and oceanographic gradients along the Atlantic–Indian Ocean transition [78], while parallel selection on visual opsin genes across fish and invertebrates has offered clues to adaptation under increasing turbidity [79,80]. Moreover, comparative analyses have revealed signatures of convergent selection on ion homeostasis and thermosensation genes in krill species [81], ancient polymorphism consistent with thermal stress resilience in corals [82,83] and proteome-level changes potentially mitigating oxidative stress in Antarctic notothenioids [84]. Similarly, comparative analyses have tested whether the efficacy of natural selection increases with effective population size (N_e_), under the assumption that N_e_ increases both the fixation of beneficial mutations and the purging of deleterious alleles [63,85]. Transcriptomic data from nine krill species support this prediction, showing that ‘*r*-selected’ species with large N_e_ have a higher proportion of adaptive substitutions fixed than ‘*K*-selected’ species with small N_e_, suggesting that adaptive potential varies with ecology and standing genetic variation [81]. In addition, global analyses of intraspecific variation across hundreds of marine fish and foundation species (e.g. corals, sponges, seagrasses and kelps) have shown that standing genetic diversity is shaped by environmental factors such as primary productivity and temperature, often declining towards the poles or showing bimodal latitudinal patterns, indicating spatial variation in adaptive potential among ecosystems [86,87].

Yet, our understanding of the ability of species to respond to rapid climate change is limited, and we lack knowledge on which biological functions are under selection across ecosystems and which intrinsic factors (e.g. life-history traits, N_e_ and genomic architecture) facilitate adaptation. This is particularly the case for foundation species and top predators, which play a crucial role in ecosystem functioning and whose responses can have cascading effects on other species they interact with.

A macrogenomics framework can fill this gap and enhance our understanding of marine ecosystem sensitivity and resilience (supplementary material, table S1). Firstly, it is unclear whether marine species can adapt fast enough to keep pace with rapid environmental change. Extending studies on the effects of catastrophic events (e.g. sticklebacks [77]) or of experimental translocations to additional species interacting in the same ecosystem could reveal how adaptive responses propagate through ecosystems. Furthermore, macrogenomic analyses combining modern, ancient and historical DNA could test cross-species synchrony in adaptive rates by detecting temporal allele frequency shifts at selected loci [15]. For example, loss of diversity in a foundation species (e.g. a coral) could cascade to dependent species such as parrotfishes through combined bottlenecks and directional selection (figure 2d).

Secondly, tests of convergent selection across phylogenetically distant taxa (e.g. algae, corals and fish) can highlight which biological functions are under the strongest selection pressures, and thus identify which functional groups and trophic levels are most critical to ecosystem resilience. Similarly, identifying hotspots of adaptive diversity for thermotolerance loci within well-documented trophic networks (e.g. kelp, sea urchin and otter [88]) could show whether adaptive variation is evenly distributed across trophic levels and geography or concentrated in particular species or locations, with important consequences for ecosystem resilience and for the designation of marine protected areas (MPAs).

Thirdly, the role that N_e_ plays in shaping adaptive responses and colonization of novel environments remains poorly understood. For instance, comparing N_e_ trajectories and signatures of selection at thermally associated loci across taxa independently exposed to the warmer, more thermally variable Mediterranean relative to Atlantic source populations could reveal whether reduced N_e_ in warm-edge populations constrains adaptive potential, or whether selection on key loci can overcome drift even in genetically depauperate populations.

Finally, how phenotypic plasticity [89], epigenetic change [90] or access to open niches and community interactions mediate adaptive responses remains poorly explored. A macrogenomic framework could compare co-occurring species with contrasting thermal plasticity (e.g. fish adapted to narrow versus broad temperature range), combining methylation data and genomic scans to assess whether epigenetic variation buffers or facilitates adaptation, or examine signatures of selection in species expanding into new habitats to reveal how novel community interactions shape the pace and targets of adaptation across a community.

### (c) Ecosystem health based on genomic variation

Genome erosion (i.e. loss of genetic diversity, increase in genetic load and maladaptation) is a major threat to population viability and biodiversity [91]. Estimates of IGV are increasingly used in conservation genomics and integrated into conservation programmes. For instance, the Global Biodiversity Framework of the Convention on Biological Diversity (www.cbd.int) outlines a monitoring framework focusing on essential biodiversity variables (EBVs), including the following indicators: effective population size (N_e_), genome-wide diversity (e.g. nucleotide diversity, heterozygosity and inbreeding), population structure and gene flow (i.e. F_ST_) [92]. Additionally, estimates of genome erosion, such as inbreeding and genetic load, are increasingly used to evaluate the genetic threats to natural populations in space and time [93].

While populations with large N_e_ and high genome-wide diversity (e.g. open-sea pelagic fish) may seem immune to genome erosion, growing evidence shows that species with high dispersal potential in open marine habitats can also lose significant genetic variation over short time periods (e.g. ponyfish [94]; Atlantic bluefin tuna [95]), likely driven by environmental change and overfishing. Furthermore, several top predators (e.g. marine mammals) critical for ecosystem functioning have experienced severe human- and climate-induced declines over the past few centuries [96], further underscoring the risk of genome erosion in marine organisms.

An early example of a macrogenetic study attempted to identify barriers to gene flow among ecologically important species from the Baltic Sea (i.e. herring, pike, whitefish, stickleback, blue mussel and bladderwrack) but found few common patterns among species [97]. Other studies showed that tropical regions generally harbour higher diversity than non-tropical ones, and human-impacted regions less than wilder ones [23], whereas small-bodied *r*-selected species show higher genetic diversity than larger, *K*-selected species [26]. By contrast, a genomic study on 17 pinniped species highlighted the predominant role of past demography, with more limited effects of ecology and life-history traits, on genome-wide variation [51]. More recent genomic studies on endangered species have shown that multi-species estimates of inbreeding or genetic load can be used to predict the risk of inbreeding depression [98] or species-specific genomic vulnerabilities [99].

Estimating these indices in a macrogenomic framework encompassing multiple trophic levels while accounting for species interactions can also be informative for monitoring biodiversity in changing environments (supplementary material, table S1). For instance, a recent study revealed synchrony between predator and prey population substructure, with gene flow among herring-feeding killer whale subpopulations being potentially reduced as a result of herring populations' collapse in the late 1960s [96]. Although temporal changes in population structure or N_e_ were not formally compared, this study illustrates the potential for estimating IGV among interacting species in a temporal framework (e.g. figure 2d).

Despite the widespread threat of genome erosion, such integrative studies remain scarce. A macrogenomic framework provides the most efficient way to monitor patterns of genome erosion among interacting species at the ecosystem level. Firstly, correlations of genomic indicators among species within trophic networks could reveal cascading effects and eco-evolutionary links between genetic processes and ecosystem dynamics. A famous example of cascading genomic effect is that of the Isle Royale moose population which expanded and averted inbreeding depression, following the near-extinction of the highly inbred Isle Royale wolf [100], though such correlations may be delayed owing to a time lag in genetic response [101]. Thus, a demographic decline of a top predator (e.g. killer whale) may lead to population growth and reduced inbreeding of a mesopredator (e.g. seal), whereas the extinction of a prey (e.g. herring) could induce an increase in inbreeding in its predator (e.g. killer whale and seal), raising the risk of secondary extinction. Additionally, increased substructuring or reduced genetic diversity in a foundation species may signal vulnerability or impending collapse of trophic networks. For instance, reduced genetic diversity in eelgrass [102] and kelp [103] is associated with lower resistance to ecological disturbance, including heatwaves, and can have cascading effects on species relying on them for habitat [104].

Secondly, the ability to estimate IGV for hundreds of species simultaneously across multiple ecosystems (e.g. California Conservation Genomics Project; https://www.ccgproject.org/) opens the possibility of developing an ecosystem-wide genetic index analogous to species or eDNA-based diversity indices [105], but focusing on genomic variation instead of species richness (e.g. [23]). Such an index could enhance biodiversity monitoring by revealing ecosystem-wide patterns of genomic diversity, including population connectivity and the strength of species interactions [22], while also providing standardized ecosystem-level measurements of genetic diversity [106]. This could be done by extending genomic metrics from the genomic green status framework [91] or EBVs [92] to the ecosystem level. However, a key challenge will be to determine how to weight the contributions of individual species, for instance, by their trophic level, interaction strength or functional importance within the ecosystem.

Ultimately, these data could inform MPA design, helping to prioritize hotspots of genomic diversity or, conversely, areas where genomic diversity is lowest and thus populations are most vulnerable to genome erosion. IGV estimates could also act as an early warning signal of ecosystem deterioration, improving our assessment of resilience in the face of climate change or the effectiveness of restoration actions [28]. Ecosystems structured by foundation species, such as corals, kelp or seagrasses, represent particularly compelling targets for such an approach, given that declines in the genomic diversity of these ecological engineers can cascade into broader losses of ecosystem function and biodiversity.

### (d) Prediction of future marine ecosystem shifts

Modelling approaches that integrate empirical genomic, environmental and distribution data are critical for predicting marine species' responses to global change, enabling inference and projection of adaptive diversity, habitat distribution, population size and species occupancy [107]. SDM using spatial climate projections [108] has been widely applied to marine taxa of economic and ecological importance, including seaweed, fish, molluscs and marine mammals to estimate range shifts in response to environmental change [17]. Such studies reveal ongoing and projected poleward range shifts in distributions driven primarily by rising temperatures, with a global loss of biodiversity in tropical areas and an increase in polar regions, leading to changes in community composition and disruption of ecosystem functions with complex socioeconomic effects [109,110]. A recent global study projected that under a high-emission scenario, nearly half of 33 000 modelled marine species will lose 50% of their core habitat area by 2100 [111].

A central challenge to predicting the impacts of climate change lies in accounting for species interactions, which can profoundly alter regional distributions and abundances and thus ecosystem function [112]. For instance, at higher latitudes, species invasions are expected to accelerate, triggering severe, yet unpredictable, changes in ecosystem structure and function, as a result of novel predator–prey and competitive interactions [111]. Joint species distribution modelling (JSDM; e.g. [3]) can generate important insights on community-level impacts. For instance, JSDM for 16 fish species in the Baltic have shown that warm-tolerant species yields have increased over the past 40 years, whereas cold-adapted species have been negatively impacted by a reduction in habitat driven by an increase in temperature and reduction in salinity [113]. However, under changing climate conditions, novel species interactions may emerge following distributional shifts, giving rise to cascading effects and ecosystem shifts that may be difficult to predict. Another challenge is that climate change will continue for hundreds of years [64], meaning that the predicted effects from models may not be representative of a state of equilibrium.

Combining SDM and genetic data has provided a robust framework for linking environmental suitability (i.e. genotype–environment association, GEA) as well as demographic, phylogeographic and adaptive processes [18,19]. For instance, seascape studies in sea scallop [114] and seagrass [115] have shown that distinguishing distinct genetic lineages and including intraspecific adaptive variation in SDM can significantly improve predictions of habitat suitability and responses to future climate change. Importantly, these approaches show that tracking adaptive variation, not just range size or N_e_, fundamentally alters climate risk predictions [18]. Moreover, genomic forecasting methods, such as fitness offset, allow the estimation of potential fitness mismatches under future conditions [20]. Comprehensive syntheses [11] highlight how these integrative methods provide mechanistic insights into evolutionary responses across spatial and temporal scales.

Additionally, genome-informed simulations (e.g. SLiM [21]) offer unprecedented power to model adaptive responses of species under global climate change with more detail than traditional landscape genomics approaches [116]. Operating at finer timescales than whole genome analyses (i.e. years/decades), SLiM can incorporate ecologically realistic parameters (e.g. life-history traits and demographic stochasticity) to model key genetic variation (i.e. adaptive and deleterious variation, and inbreeding) and processes (i.e. gene flow and selection), and can be applied to real-world conservation scenarios such as assessing the impacts of harvesting on genome-wide variation, estimating fitness offset and predicting the loss of adaptive variation under rapid environmental change [117]. SLiM can also be used in a spatial framework to take into account population connectivity (e.g. [118]), by using abundance projections from JSDM as a proxy for population size for multiple interacting species ( figure 3).

**Figure 3.**
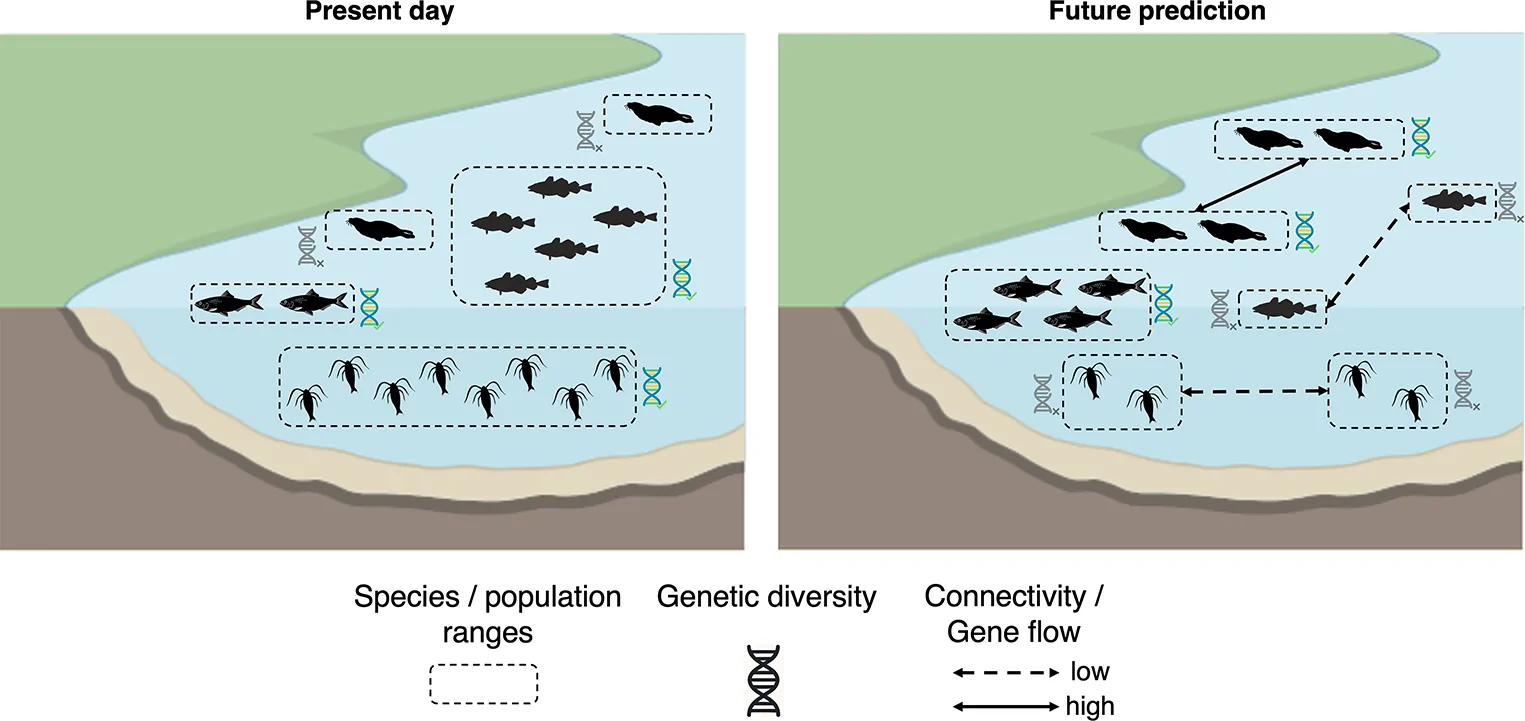
Usage of spatially explicit genome-informed simulations to predict future changes in marine ecosystems. In this hypothetical example, genomic variation is simulated within a spatial framework, based on future species distribution ranges inferred with SDM. Cod populations decline owing to sea warming and overfishing. As a predator of herring, their decline allows herring populations to increase, which initially supports growth and connectivity among grey seal populations (a top predator). However, rising temperatures will also reduce zooplankton (e.g. copepod) populations, potentially driving declines in herring, cod and grey seal populations, alongside reduced connectivity, gene flow and genetic diversity. Coloured and grey DNA helices depict high or low genetic diversity, respectively.

Combining future distribution projections with genome-informed simulations in a macrogenomic framework represents the most promising approach to predict ecosystem-wide responses to climate change [27]. Yet, its rare implementation leaves a critical gap in our understanding of how species interactions, genetic diversity and range shifts will jointly reshape ecosystems under future climate scenarios.

A powerful application of macrogenomics lies in its capacity to generate community-wide vulnerability maps through species-specific GEA analyses conducted along environmental gradients (e.g. temperature). By synthesizing GEA results across species within a community, it becomes possible to identify which populations harbour high or low genetic variation at thermally associated loci. Such a cross-species comparison connects to broader seascape genomic frameworks in which mismatches between current genomic makeup and projected environmental conditions flag the most vulnerable units for management.

From a conservation perspective, combining community-scale GEA and simulations supports ecosystem-based management by refining harvesting strategies, guiding habitat restoration and informing MPA delineation and prioritization. Hotspots of adaptive genetic diversity (i.e. regions where multiple species co-occur with high variation at environmentally associated loci) are prime candidates for protection, as conserving them preserves the evolutionary potential communities need to persist under climate change. IGV and locally adapted genotypes inferred from spatial analyses should therefore be explicit targets in MPA design. By mapping these attributes across assemblages simultaneously, macrogenomics offers a principled basis for spatially explicit biodiversity targets and embedding evolutionary potential into marine conservation planning.

## 4. Summary and future directions

In this review, we demonstrate how macrogenomics can be used to study and forecast marine ecosystem shifts under global climate change using empirical genomic data and predictive approaches. Although macrogenomics is still in its early stages, with most large-scale syntheses to date relying on a few loci rather than whole-genome data, the transition towards genomic-scale approaches is already underway, and its implications for the management of marine resources are becoming increasingly clear. Importantly, while each approach discussed here presents valuable but partial insights into how marine ecosystems respond to global change, their integration within a macrogenomic framework provides a comprehensive basis for developing effective conservation and management strategies.

Nevertheless, realizing the potential of macrogenomics for marine ecosystems faces several technical challenges. Firstly, it will be essential to increase efforts to quantify IGV across the broad diversity of marine life, prioritizing ecologically important but often neglected taxa from lower trophic levels, such as invertebrates, macroalgae and phytoplankton, which remain underrepresented in genomic databases relative to economically important fish and flagship species like marine mammals [119]. Additionally, it will be crucial to expand genomic sampling beyond the temperate waters of the Northern Hemisphere that have dominated the field, prioritizing understudied species from the Southern Hemisphere and tropical regions that are highly vulnerable to global warming [94,119]. Sequencing initiatives targeting underrepresented taxa and biogeographic regions, potentially through international consortia and coordinated sampling efforts, could help address these taxonomic and geographic gaps.

Secondly, expanding genomic resources, from reference genomes and gene annotations to population-level genomic data and metagenomic databases, across underrepresented and non-model taxa remains critical, as geographic and taxonomic gaps limit population-level and community-wide analyses [105,120,121]. Collaborative frameworks prioritizing genome assembly and standardized reference databases for ecologically important species would directly address this.

Thirdly, improving recovery of high-quality DNA from degraded historical and ancient marine samples, including sediments, will be essential, particularly for species with fragmented fossil records, since even rare specimens can be highly informative [16]. Advances in aDNA and sedaDNA approaches optimized for marine environments would thus contribute crucial insights into past changes in marine biodiversity [27]. Yet, metagenomic data from both modern and ancient material still present biases and limitations, particularly regarding species detection and the correlation between read counts and biomass, and eDNA is further subject to variable environmental stress and degradation rates [105,120].

Fourthly, while data from a few species (e.g. keystone predator, mesopredator and foundation species) may be sufficient to predict major ecosystem shifts, the complexity of trophic interactions across a broader range of species can make future predictions more challenging. Furthermore, predicting future ecosystem shifts and species interactions is particularly difficult, especially as transient and novel interactions may emerge under climate change [111]. Integrating trophic interactions, experimental data and genomics could thus help model novel complex multi-species interactions and validate predictions under different climate change scenarios.

Fifthly, a key limitation of macrogenomics is that the scope will vary depending on the data available (i.e. type, temporal and geographic resolution) and the method used. For instance, only a single genome is required to capture deep-time demographic history for some SMC methods, whereas population-level data are required to resolve more recent fluctuations. Therefore, no single dataset or method can address all questions, and integrating multiple data types may be necessary to examine biodiversity change across timescales.

Finally, the thorough archiving of genetic data and associated sample metadata, together with open access to accurate taxonomic and oceanographic databases, is essential for providing marine macrogenomics with the information and insights needed to address the challenges posed by climate change [122]. Adoption of FAIR data principles [123], including harmonization of sampling protocols and bioinformatic pipelines across ecosystems and organismal groups, would ultimately facilitate the integrative, cross-scale analyses that will be essential to anticipate and monitor biological responses to global climate change. Embedding these principles into macrogenomic practice from study design onward, rather than retrospectively, will be key to unlocking the cumulative value of distributed sampling efforts and translating genomic data into actionable biodiversity forecasts.

## Supplementary Material



## Data Availability

This study is based on publicly available records of NGS libraries from the NCBI Sequence Read Archive (SRA) and the European Nucleotide Archive (ENA) downloaded using the ENA Browser API on 26 August 2025 and available in the Zenodo repository [124]. Names of marine species were retrieved from the World Register of Marine Species (WoRMS) [29] and Listing the World's Algae [30]. Owing to terms of usage and agreements necessary to access these comprehensive taxonomic datasets, redistribution of the full taxonomic lists to third parties is restricted. Only the subset of NGS records matching marine species names were analysed, and these records are available in the Zenodo repository [124], along with the code and raw data used to generate species statistics (figure 1, and figure S1 in the supplementary material). Supplementary material is available online [125].
