# Supplementary material for: Harnessing macrogenomics to study changing marine ecosystems under global climate change
Source: Proc Biol Sci. 2026 Oct 7;293(2080):20260357. doi: 10.1098/rspb.2026.0357 (PMC13641918; doi:10.1098/rspb.2026.0357)
Supplement: Supplementary file 1 [file rspb.2026.0357.zip › rspb20260357_si_001.pdf]

Electronic Supplementary Material for :

Harnessing macrogenomics to study changing marine ecosystems under global climate change

Nicolas Dussex[1,*], Leif Andersson[2,3], Ulf Bergström[4], Kerstin Johannesson[5], Andreas Wallberg[2]

1. Department of Population Analysis and Monitoring, Swedish Museum of Natural History, SE-106 91 Stockholm, Sweden
2. Department of Medical Biochemistry and Microbiology, Uppsala University, Uppsala, Sweden
3. Department of Veterinary Integrative Biosciences, Texas A&M University, College Station, USA
4. Department of Aquatic Resources, Swedish University of Agricultural Sciences, Uppsala, Sweden
5. Department of Marine Sciences, University of Gothenburg, Tjärnö Marine Laboratory, SE-452 96 Strömstad, Sweden

*corresponding author: nicolas.dussex@gmail.com; Nicolas.Dussex@nrm.se

DOI: 10.1098/rspb.2026.0357

Methods used to compile NGS sample and species statistics

The quantitative data used to compile statistics for Figure 1 and Supplementary Figure 1 was downloaded from multiple sources. First, the Global Core Biodata Resource the European Nucleotide Archive (ENA) was accessed through its public ENA Browser API on 2025-08-26 using the program curl in order to download “sample”, “read_run” and “study” records for genomic data into three separate and extensive TSV files, respectively. Commands:

To download runs (12 GB TSV file):

curl --retry 5 --retry-delay 10 --keepalive-time 60 -X POST "https://www.ebi.ac.uk/ena/portal/api/search" -d "result=read_run" -d "fields=run_accession,sample_accession,experiment_accession,study_accession,tax_id,scientific_name,library_strategy,library_source,instrument_platform,country,collection_date,lat,lon,location,marine_region,first_created,project_name,study_title,sample_description" -d "limit=0" -d "offset=0" -d "format=tsv" > ena_runs.description.tsv

To download samples (18 GB TSV file):

curl --retry 5 --retry-delay 10 --keepalive-time 60 -X POST "https://www.ebi.ac.uk/ena/portal/api/search" -d "result=sample" -d "fields=submission_accession,sample_accession,secondary_sample_accession,tax_id,scientific_name,tax_lineage,sample_title,project_name,description,sample_description" -d "limit=0" -d "offset=0" -d "format=tsv" > ena_samples.description.tsv

To download studies (1 GB TSV file):

curl --retry 5 --retry-delay 10 --keepalive-time 60 -X POST "https://www.ebi.ac.uk/ena/portal/api/search" -d "result=study" -d

```
"fields=study_accession,description,first_public,keywords,project_name,study_name,study_title,stud
y_description"     -d "limit=0"     -d "offset=0"     -d "format=tsv" > ena_study.description.tsv
```

These records contain a snapshot of all public genomic data shared between ENA and the NCBI Short Read Archive (SRA) at that time, including n=45,940,433 samples, 37,691,758 runs and 923,415 studies. Second, taxonomic records for valid scientific names and classifications of marine species were downloaded from the World Register of Marine Species (WoRMS) database after acquiring login and permissions by the curators. In addition, a subset of AlgaeBase records for valid scientific names of marine plants and algae matching those with ENA records were provided by database curators. Non-redundant lists of valid scientific names with or without classifications (kingdom, phylum, class and order) were created, spanning in total 239,346 marine species. Due to terms of usage and agreements necessary to access these comprehensive taxonomic datasets, re-distribution of full taxonomic lists to third parties is restricted.

Next the genomic data records and taxonomic lists were processed and cross-referenced with custom Perl scripts. First, multiple fields in both the "study" and "sample" records were queried using regular expression in case insensitive mode to match any of several keywords to detect possible ancient DNA datasets:

aDNA
ancient\W+DNA
sedaDNA
sedimentary\sancient\W+DNA
paleogenomics
museum\ssample
museum\sspecimen

Likewise, metagenomic data was detected using regular expression. First, "study" and "sample" records were searched for matches against:

marine
seawater

Second, multiple fields were additionally searched for mentions of:

metagenom
metatranscriptom

Additionally, the scientific name of a record either had to match a valid species perfectly or any of the following keywords commonly found in that field for metagenomic data:

marine sediment
marine meta
marine plankton
seawater
coral
sponge
alga
sea
seagrass
hydrozoan
beach
sand
mangrove
shrimp

oyster
fish
invertebrate
hydrothermal
cold seep
mollusc
gill
jellyfish
ballast
biofouling
volcano
ice
estuary

However, an additional filter was applied to remove any potential metagenomic records if they also mentioned *human*, *Homo*, *Mus* or *indoor* in the name field.

By requiring a match against a valid marine scientific name for a sample record or matching it against keywords for marine metagenomes, as well as considering it to be ancient DNA or not, the marine genomic sample records were partitioned into four groups:

Number of sample records	contemporary origins (n)	ancient/historical origins (n)
an individual, single-species sample	428,347	1,809
a metagenome sample	230,444	1,004

These sample records were additionally annotated with fields from the runs and studies they were part of (such as sequencing source and strategy) and exported as separate TSV tables provided as Supplementary data 1–4. In addition, these samples were counted on a per-species basis, whereby samples including the following field information were considered to have geographic information:

- existence of latitude or longitude coordinates
- existence of location of origin
- existence of country of origin

These fields tended to separate samples collected from natural populations from those produced in experimental settings. We also performed geolocation of the contemporary/modern data libraries using an R script that first classified samples with available latitude/longitude by spatial intersection or nearest-distance matching to global marine polygons, then attempted fallback classification from coordinates embedded in text fields, explicit marine/coastal place-name rules, US state or territory rules, country-level rules, and finally cached Nominatim geocoding for unresolved locality strings. The workflow used Tidyverse for data handling, Simple Features for R together with Natural Earth spatial data for marine polygon operations, Country code for country-name normalization and Tidygeocoder for optional cached geocoding of remaining free-text locations. This resulted in 181,785 geolocated records.

Moreover, the following sequencing strategies and sources were used to partition samples by library technology:

Type of library	Strategy (case insensitive)	Source (if used)
Whole-Genome Sequencing (WGS)	*wgs* or *wga*	*GENOMIC*
Transcriptomic (RNA-seq)	*RNA-seq*	*TRANSCRIPTOMIC*
Whole exome sequencing (WXS)	*wxs*	*GENOMIC*
Amplicons	*AMPLICON*	
Restriction-site Associated DNA sequencing (RAD-seq)	*RAD\W+Seq*	

The different kinds of sample libraries were counted for both species-level and metagenomic data. Whole exome sequencing libraries were so few overall compared to other libraries that they were not reported in the main data figure. The per-species sample library counts are provided for each of the four groups of data (species-contemporary; species-ancient; metagenomic-contemporary; metagenomic-ancient) as Supplementary data 5–8.

Using a separate script, the species-level library counts were partitioned by informal organism groups (*ray-finned, fishes, cartilaginous fishes, invertebrates, plants and algae, birds, other reptiles, mammals* or *other chordates*) based on taxonomy. The number of species with a number of samples derived from different library technologies that reached certain thresholds (more than 1, 10 or 50) were counted and are provided as Supplementary Data 9 and 10 for contemporary and ancient datasets, respectively.

The corresponding metagenomic counts were assessed by eye and partitioned into environmental (e.g. from a marine sediment or water column) or organismal (e.g. from the gut of a host organism) groups. A table merging counts for both contemporary and ancient libraries are provided as Supplementary Data 11.

The sample libraries were also assessed for the year (between 2010 and 2025) at which they or the run or study were created in ENA/SRA. An overall timeline table for the four groups of data (species-contemporary; species-ancient; metagenomic-contemporary; metagenomic-ancient) is provided as Supplementary Data 12.

Lastly, the overall and final counts of marine species partitioned by informal organism groups (see above) based on taxonomy and the corresponding subset counts of species with any sequencing data are provided as Supplementary Data 13. These overall counts (column “GROUP_SIZE”) for the different informal groups were adjusted slightly to account for minor discrepancies involving synonyms or invalid names emerging at different timepoints during the process of finalizing the species list (from n=239,052 species names in total to n=239,346 overall).

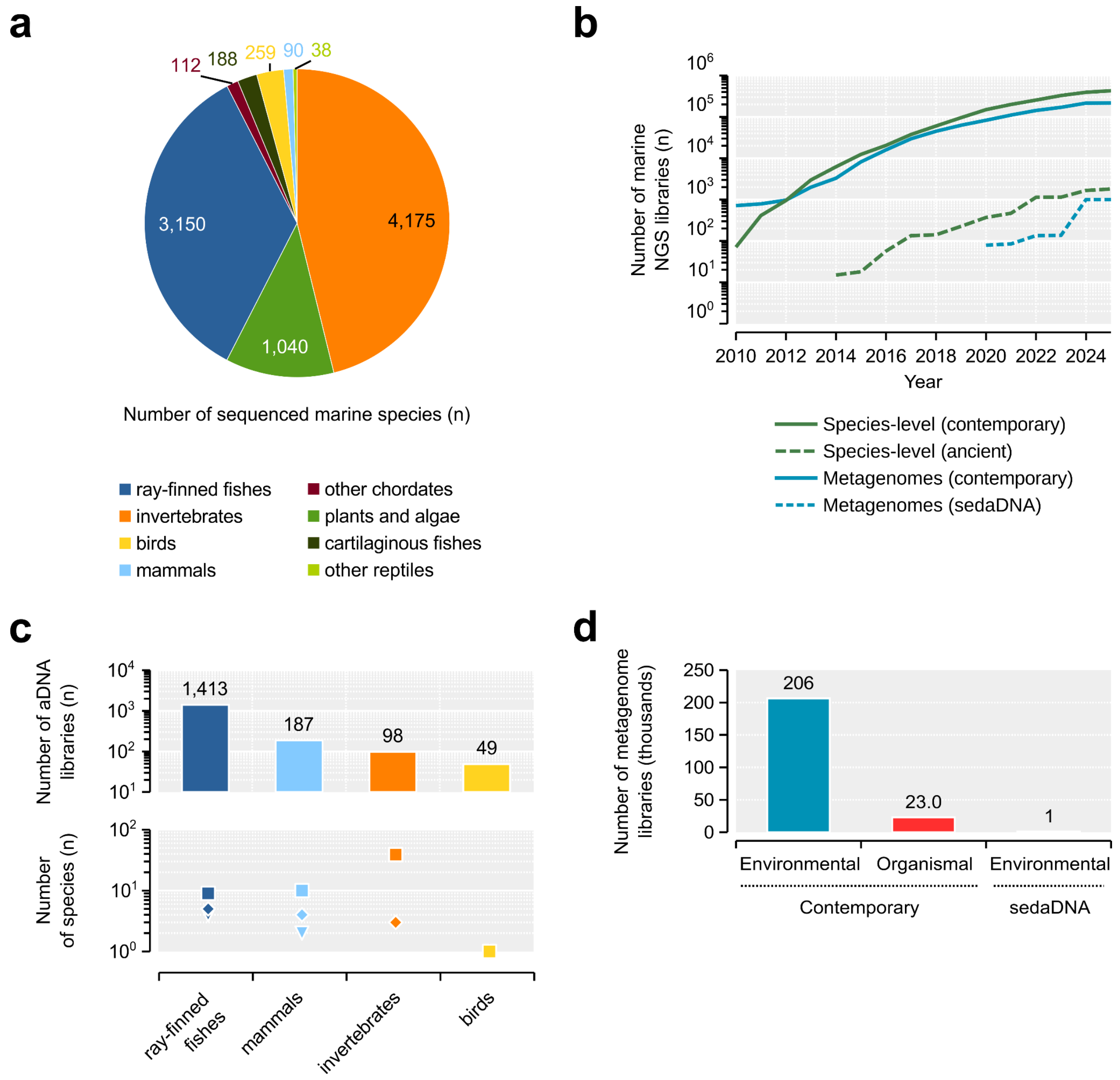

Supplementary Figure 1. Published marine genomic NGS libraries (modern and ancient samples). NGS libraries in the NCBI Sequence Read Archive (SRA) or the European Nucleotide Archive (ENA). **a.** Taxonomic representation of NGS data. **b.** Accumulated temporal increase in NGS libraries. Ancient = historical or ancient DNA (aDNA). As of 2025-08-26, there are >420,000 NGS libraries from contemporary marine specimens, >1,800 aDNA libraries and >230,000 metagenomic libraries deposited in the public NCBI Sequence Read Archive (SRA) or the European Nucleotide Archive (ENA). **c.** Upper: number of aDNA WGS libraries per group. Lower: number of species with n≥1 library (i.e., comparative genomics), n≥10 libraries (i.e., intraspecific levels of variation) or n≥50 aDNA libraries (i.e., population structure and adaptive variation). **b.** Number of marine metagenomes per environment type. Organismal = gut content, Environmental = water or sediments.

Supplementary Table 1. Examples of datasets that could be combined in macrogenomics and of potential applications for marine studies. Predictive approaches which are invaluable complements to empirical data and which can be applied in a macrogenomics framework are also mentioned.

**Question**	**Analytical approach**	**Data type**	**Examples of available datasets and region**
How sensitive were species to past climatic and environmental fluctuations (i.e., 100s of 1000s to millions of years Before Present)?	Coalescent (e.g., PSMC, SMC) linkage disequilibrium approaches	WGS - a single or multiple genomes per population)	Pinnipeds - worldwide [1]<br>Rorqual whales - worldwide [2]
How were species communities impacted by past climatic and environmental fluctuations?<br><br>Can we track shifts in distributions and invasive species?	Metagenomics to detect species presence/absence and abundance	eDNA, sedaDNA	Crabs and green algae - Arctic [3]<br>Eukaryotes - Antarctic [4]<br>Penguins - Antarctic [5]<br>Eukaryotes, prokaryotes, fish - Arctic [6]<br><br>Invertebrates - North Atlantic [7]<br>Invertebrates, Vertebrates - North Atlantic [8]<br>Fish - Antarctic [9]<br>Eukaryotes, barnacles - North Sea and Baltic Sea [10]
How can past signatures of adaptation help us predict future adaptive responses to climatic and environmental change?	Temporal genomics, genome-environment correlations, tests of selection	Population-level WGS, aDNA, histDNA, targeted gene sequencing	Herring - Atlantic and Baltic Sea [11–13]<br>Cod - Atlantic and Baltic [14]<br>Spratt - North, Baltic and Black Sea [15]<br>Thorny Skate - North Atlantic [16]<br>Pacific Oyster - Yellow Sea [17]
How can adaptation along environmental gradients or among distinct environments predict future adaptive responses to climatic and environmental change?<br><br>Can hotspots of adaptive variation across species within an ecosystem be identified?	Temporal genomics, space-for-time approaches	Population-level WGS or RNA-seq, targeted gene sequencing, multi-species comparative data	Vertebrate (e.g. fish), invertebrates and unicellular organisms, algae - Baltic [18]<br>Krill - Arctic, Antarctic [19,20]<br>Invertebrates - Indian Ocean [21]
How have past climatic and environmental changes impacted genome erosion	Temporal genomics, IGV	Population-level WGS, targeted gene sequencing	Ponyfish - Philippine Sea [22]<br>Atlantic tuna - Mediterranean [23]<br>Killer whale - North Atlantic [24]

patterns within ecosystems?			
How will changing environmental conditions shape the spatial distribution of genomic diversity and local adaptation? How maladapted will populations be to future conditions?	Species Distribution Models (SDM); genotype–environment association (GEA) analyses; genomic offsets	Occurrence data, genomic data, environmental variables (e.g., sea surface temperature, salinity) used as predictors in GEA	Bio-Oracle marine data layers - worldwide [25]<br><br>Sea scallop - North Atlantic [26]<br>Seagrass [27]
How will genome-wide variation be affected by future climatic and environmental change?	Genome-informed simulations (e.g., SLiM)	Current estimates of IGV, life-history and ecological parameters, future projections of species abundance and distribution from SDM	Killer whale - North Atlantic [24]<br>Cod - Atlantic and Baltic [14]<br>Salmon - North Atlantic [28,29]<br>Coral reefs - worldwide [30]

References

1. Peart CR *et al.* 2020 Determinants of genetic variation across eco-evolutionary scales in pinnipeds. *Nat Ecol Evol* **4**, 1095–1104.

2. Árnason Ú, Lammers F, Kumar V, Nilsson MA, Janke A. 2018 Whole-genome sequencing of the blue whale and other rorquals finds signatures for introgressive gene flow. *Sci. Adv.* **4**, eaap9873.

3. Kjær KH *et al.* 2022 A 2-million-year-old ecosystem in Greenland uncovered by environmental DNA. *Nature* **612**, 283–291.

4. Armbrecht LH. 2020 The potential of sedimentary ancient DNA to reconstruct past ocean ecosystems. *Oceanography (Wash. D. C.)*

5. Wood JR *et al.* 2025 Sedimentary DNA insights into Holocene Adélie penguin (Pygoscelis adeliae) populations and ecology in the Ross Sea, Antarctica. *Nat Commun* **16**, 1798.

6. Zimmermann HH, Stoof-Leichsenring KR, Dinkel V, Harms L, Schulte L, Hütt M-T, Nürnberg D, Tiedemann R, Herzschuh U. 2023 Marine ecosystem shifts with deglacial sea-ice loss inferred from ancient DNA shotgun sequencing. *Nat. Commun.* **14**, 1650.

7. Gallego R *et al.* 2024 North Atlantic deep-sea benthic biodiversity unveiled through sponge natural sampler DNA. *Commun Biol* **7**, 1015.

8. Serivichyaswat PT, Scholte T, Wilms T, Stranddorf L, van der Valk T. 2025 Metagenomic biodiversity assessment within an offshore wind farm. *Sci Rep* **15**, 16786.

9. Jeunen G-J *et al.* 2024 Unlocking Antarctic molecular time-capsules - Recovering historical environmental DNA from museum-preserved sponges. *Mol Ecol Resour* **24**, e14001.

10. Junk I, Schmitt N, Krehenwinkel H. 2023 Tracking climate-change-induced biological invasions by metabarcoding archived natural eDNA samplers. *Curr. Biol.* **33**, R943–R944.

11. Atmore LM *et al.* 2024 The Once and Future Fish: Assessing a Millennium of Atlantic Herring Exploitation Through Mixed-Stock Analysis and Ancient DNA. *Glob Chang Biol* **30**, e70010.

12. Han F *et al.* 2020 Ecological adaptation in Atlantic herring is associated with large shifts in allele frequencies at hundreds of loci. *Elife* **9**. (doi:10.7554/eLife.61076)

13. Goodall J *et al.* 2024 Evolution of fast-growing piscivorous herring in the young Baltic Sea. *Nat. Commun.* **15**, 10707.

14. Pinsky ML *et al.* 2021 Genomic stability through time despite decades of exploitation in cod on both sides of the Atlantic. *Proc. Natl. Acad. Sci. U. S. A.* **118**, e2025453118.

15. Pettersson ME *et al.* 2024 Limited parallelism in genetic adaptation to brackish water bodies in European sprat and Atlantic herring. *Genome Biology and Evolution* **16**, evae133.

16. Lesturgie P *et al.* 2025 Short-term evolutionary implications of an introgressed size-determining supergene in a vulnerable population. *Nat. Commun.* **16**, 1096.

17. Hu B, Tian Y, Li Q, Liu S. 2022 Genomic signatures of artificial selection in the Pacific oyster,. *Evol Appl* **15**, 618–630.

18. Johannesson K, Le Moan A, Perini S, André C. 2020 A Darwinian laboratory of multiple contact zones. *Trends Ecol. Evol.* **35**, 1021–1036.

19. Choquet M, Lenner F, Cocco A, Toullec G, Corre E, Toullec J-Y, Wallberg A. 2023 Comparative population transcriptomics provide new insight into the evolutionary history and adaptive potential of world ocean krill. *Mol. Biol. Evol.* **40**. (doi:10.1093/molbev/msad225)

20. Unneberg P *et al.* 2024 Ecological genomics in the Northern krill uncovers loci for local adaptation across ocean basins. *Nat Commun* **15**, 6297.

21. Nielsen ES, Henriques R, Beger M, Toonen RJ, von der Heyden S. 2020 Multi-model seascape genomics identifies distinct environmental drivers of selection among sympatric marine species. *BMC Evol. Biol.* **20**, 121.

22. Clark RD *et al.* 2025 Anthropocene genetic diversity loss in the marine tropics. *Proc Natl Acad Sci U S A* **122**, e2513012122.

23. Andrews AJ *et al.* 2025 Ancient DNA suggests a historical demographic decline and genetic erosion in the Atlantic bluefin tuna. *Proc. Natl. Acad. Sci. U. S. A.* **122**, e2409302122.

24. Baumgartner CD *et al.* 2025 Kinship clustering within an ecologically diverse killer whale metapopulation. *Heredity (Edinb.)* **134**, 109–119.

25. Assis J, Tyberghein L, Bosch S, Verbruggen H, Serrão EA, De Clerck O, Tittensor D. 2018 Bio-ORACLE v2.0: Extending marine data layers for bioclimatic modelling. *Glob. Ecol. Biogeogr.* **27**, 277–284.

26. Lowen JB, Hart DR, Stanley RR, Lehnert SJ, Bradbury IR, DiBacco C. 2019 Assessing effects of genetic, environmental, and biotic gradients in species distribution modelling. *ICES Journal of Marine Science* **76**, 1762–1775.

27. Hu Z-M *et al.* 2021 Intraspecific genetic variation matters when predicting seagrass distribution under climate change. *Mol. Ecol.* **30**, 3840–3855.

28. Levis NA, Pfennig DW. 2016 Evaluating 'Plasticity-First' Evolution in Nature: Key Criteria and Empirical Approaches. *Trends Ecol Evol* **31**, 563–574.

29. Kess T *et al.* 2024 Variable parallelism in the genomic basis of age at maturity across spatial scales in Atlantic Salmon. *Ecol Evol* **14**, e11068.

30. Pinsky ML, Clark RD, Bos JT. 2023 Coral reef population genomics in an age of global change. *Annu. Rev. Genet.* **57**, 87–115.
